# Differential Effects of Superoxide Dismutase Mimetics after Mechanical Overload of Articular Cartilage

**DOI:** 10.3390/antiox6040098

**Published:** 2017-11-30

**Authors:** Mitchell C. Coleman, Marc J. Brouillette, Nicholas S. Andresen, Rebecca E. Oberley-Deegan, James M. Martin

**Affiliations:** 1 Department of Orthopedics and Rehabilitation, University of Iowa, Iowa City, IA 52242, USA; marc-brouillette@uiowa.edu (M.J.B.); nick-andresen@uiowa.edu (N.S.A.); james-martin@uiowa.edu (J.M.M.); 2 Department of Biochemistry and Molecular Biology, University of Nebraska Medical Center, Omaha, NE 68198, USA; becky.deegan@unmc.edu

**Keywords:** superoxide, superoxide dismutase (SOD), arthritis, trauma, mitochondria, cartilage

## Abstract

Post-traumatic osteoarthritis can develop as a result of the initial mechanical impact causing the injury and also as a result of chronic changes in mechanical loading of the joint. Aberrant mechanical loading initiates excessive production of reactive oxygen species, oxidative damage, and stress that appears to damage mitochondria in the surviving chondrocytes. To probe the benefits of increasing superoxide removal with small molecular weight superoxide dismutase mimetics under severe loads, we applied both impact and overload injury scenarios to bovine osteochondral explants using characterized mechanical platforms with and without GC4403, MnTE-2-PyP, and MnTnBuOE-2-PyP. In impact scenarios, each of these mimetics provides some dose-dependent protection from cell death and loss of mitochondrial content while in repeated overloading scenarios only MnTnBuOE-2-PyP provided a clear benefit to chondrocytes. These results support the hypothesis that superoxide is generated in excess after impact injuries and suggest that superoxide production within the lipid compartment may be a critical mediator of responses to chronic overload. This is an important nuance distinguishing roles of superoxide, and thus superoxide dismutases, in mediating damage to cellular machinery in hyper-acute impact scenarios compared to chronic scenarios.

## 1. Introduction

The healthcare burden of post-traumatic osteoarthritis (PTOA) upon the United States is significant with a disproportionate number of young and active people suffering this disease as a result of trauma to joints in the lower extremities [1,2,3]. A subset of these patients that suffer severe injuries like intraarticular fractures will progress to radiographically apparent disease by four years, a grim prognosis for young patients who will not be viable candidates for joint replacement for 20–30 years [4,5]. Even for less severe injuries like meniscal injuries, ligament tears, and dislocations, there are no currently available treatments to subvert development of disease later in life. Given this societal burden and lack of viable therapies prior to joint replacement, new approaches to preventing and treating PTOA are sorely needed.

Clinical studies attempting to decrease incidence of PTOA to date have broadly focused upon improvements in surgical care [6,7,8,9] or anti-inflammatory strategies [10]. Many examinations of PTOA focused on improving surgical care have noted strong correlations between increased mechanical stresses and strains from injury to articular cartilage and subsequent PTOA development [11,12,13,14]. Benchtop efforts at explaining this clinical observation suggest the mechanical responses of chondrocytes within articular cartilage include oxidant production that can lead to injury when exceeding normal levels. Lee and Urban noted that the normal chondrocyte anabolic functions responsible for maintaining cartilage ceased in anoxia and would resume with the addition of exogenous electron acceptors, i.e., oxidants [15,16]. Terkeltaub et al., showed that these anabolic responses to normal loads were dependent upon electron transport by the mitochondria and that mitochondrial function could modulate the effects of nitric oxide and inflammation on chondrocytes [17,18]. We have previously shown that inhibition of electron transport in vitro using rotenone, or the addition of exogenous antioxidants such as N-acetylcysteine (NAC), halts this response to normal mechanical load [19,20]. After severe impacts, oxidant production sharply increases and prevention of the resultant cell death can be achieved through antioxidant supplementation via NAC or Mito-Q as well as through inhibition of the source of reactive oxygen species (ROS), the mitochondria, with rotenone [19,21]. These studies have led to our general hypothesis that the normal oxidative metabolism of chondrocytes could give rise to a mitochondria-dependent cellular injury after mechanical impact through overstimulation of the responsive pathways.

Oxidative damage and oxidative stress can also be induced by repeated overloading in the absence of an impact [22,23]. Traumatic injuries are often compounded by any resulting abnormal mechanics, for example as a result of joint incongruity in the case of intraarticular fractures or joint instability after meniscal injuries or ligament tears. Repeated mechanical overload in vitro causes a marked decrease in the mitochondrial antioxidant manganese superoxide dismutase (MnSOD) [23] despite noted increases in oxidative damage, a paradox recreated by chondrocytes taken from severely arthritic knees where MnSOD is low but oxidative stress is high [24,25,26]. Taken together, many studies have suggested that mitochondrial superoxide has a role to play at different phases of both PTOA and primary osteoarthritis (OA) development. However, mechanistic descriptions of the effects of increasing superoxide removal after well-characterized and controlled mechanical injuries are needed.

We wanted to explore the effect of increasing superoxide removal after mechanical injury without the use of native enzymes or transgenic modifications that might perturb otherwise healthy chondrocytes in situ. Therefore, we applied three different superoxide dismutase (SOD) mimetics to two previously characterized models of mechanical injury utilizing osteochondral sections with the articular surface intact. The first mimetic applied, GC4403 (formerly M40403) [27], is highly specific for superoxide, freely crosses the plasma membrane, and is present primarily in the aqueous compartment of the cell. The second compound, MnTE-2-PyP (T2E) [28], is comparable to GC4403 in its ability to enter cells and its aqueous localization but has a slightly lower rate constant for superoxide and relies upon a different chemical structure and activity to achieve superoxide dismutation. The last compound, MnTnBuOE-2-PyP (BuOE) [29], is a lipophilic analog of T2E that will localize to lipid compartments within the cell. These mimetics were preloaded into osteochondral explants 1 h prior to mechanical overload and endpoints for cell viability, mitochondrial content, and mitochondrial function were assessed 24 h later to determine each mimetic’s success or failure in preventing chondrocyte death or perturbation of mitochondrial homeostasis.

## 2. Materials and Methods

### 2.1. Tissue Harvest and Culture

Bovine stifle joints from 2–3 year old agricultural cattle were obtained from a local abattoir (Bud’s Custom Meats, Riverside, IA, USA) with the synovia intact. No instances of osteoarthritis were observed in these animals. For impact loading scenarios, matched 12 mm diameter osteochondral explants were cut from the loaded portion of the femoral condyle. Only cartilage from the weight bearing region of either condyle was used and the medial and lateral explants were evenly distributed among different treatments. Thus, for each bovine specimen obtained and four directly adjacent explants from one compartment would be randomly distributed to Control, GC4403, T2E, or BuOE groups and this matched set would then be treated with the same concentrations of the appropriate compound (or sham treatment for controls). Unloaded portions of cartilage were not utilized for this study given recent findings that unloaded cartilage has distinct responses to mechanical load [30]. For chronic loading scenarios, matched 12 × 12 mm osteochondral explants were taken from meniscus-uncovered, adjacent loaded areas of the tibial plateau from either the medial or lateral compartment. These explants have been described in greater detail and optimized for each system previously [19,20,21,22]. Explants were cultured in 45% DMEM, 45% F-12, 10% fetal bovine serum, 100 U/mL penicillin, 100 μg/mL streptomycin, and 2.5 μg/mL amphotericin B (All Gibco) in incubators maintained at 37 °C, 5% CO_2_, and 5% O_2_.

### 2.2. SOD Mimetic Treatments

**Figure 1:**
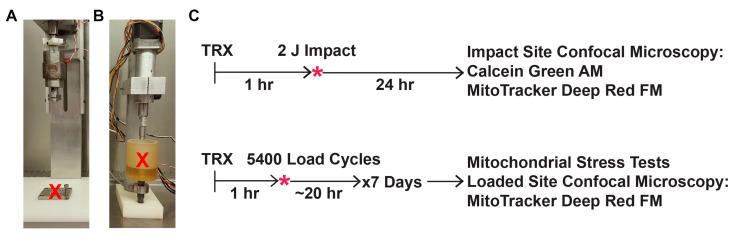
Schematic of drop tower and repetitive compressive systems and experimental design. (**A**) Pictures of the drop tower and (**B**) axial compression system are marked with a red “X” to indicate where explants sit underneath the loading platens. (**C**) All explants were subjected to a loading regimen, either a single impact or seven days of repeated loading, given 24 h to recover, and then harvested for all endpoints.

SOD mimetics were a generous gift from Galera Therapeutics (Malvern, PA, USA) for GC4403 and from Dr. James Crapo at National Jewish Health (Denver, CO, USA) for T2E and BuOE. Treatments were applied 1 h prior to loading to allow permeation of the entire articular depth. Treatment groups in the impact setting included: 100 nM, 250 nM, 500 nM, or 1 μM GC4403; 100 nM, 250 nM, 500 nM, or 1 μM T2E; or 100 nM, 250 nM, 500 nM, or 1 μM BuOE. Higher doses of these compounds were not recommended by the literature and proved of no benefit in pilot studies. This dose response was used to determine an optimal dose for comparison of the two agents, GC4403 and BuOE, utilized in the chronic loading where throughput is significantly lower than the impact setting. Increases in SOD activity were measured in primary chondrocytes plated at a high density and exposed to each concentration of mimetic for 24 h and scrape harvested. Increases in activity were confirmed using the Oberley/Spitz method which relies upon competitive inhibition of the reduction of nitroblue tetrazolium by superoxide generated via xanthine/xanthine oxidase [31]. Treatment groups exposed to repeated mechanical overload received either 1 μM GC4403 or 1 μM BuOE only during the axial loading sessions. Loading rigs and experimental design can be seen in Figure 1.

### 2.3. Mechanical Impact Delivered via Drop Tower

Following two days of equilibration to culture conditions, explants were pretreated with each mimetic or sham treatment for 1 h and then stably potted onto 3 × 3 cm stainless steel plates using polycaprolactone [19,20,21]. Reproducible 2 J impacts were delivered via custom drop tower tipped by a 5.5 mm flat, impermeable, beveled, brass platen (Figure 1A). This magnitude was chosen because in this system it induces a modest level of cell death immediately upon impact that increases over the subsequent 24 h as a result of oxidative damage [19,20,21]. After impact, samples were immediately returned to culture including SOD mimetic treatment for 24 h.

### 2.4. Mechanical Overload Delivered via Repeated Axial Compression

Following two days of equilibration to culture conditions, explants were stably potted into a custom loading apparatus housed within a tissue culture incubator (Figure 1B). Then, under culture conditions (5% O_2_ and 5% CO_2_), explants were subjected to 0.25 MPa or 1.0 MPa for 5400 cycles (about three hours) at a frequency of 0.5 Hz applied via an 8 mm flat, impermeable, stainless steel, beveled platen daily for one week as previously described [22]. The 0.25 MPa load was chosen to correspond to a healthy, control repeated loading while the 1.0 MPa load was chosen because it does not decrease cell viability but it does induce oxidative stress and dysfunction of mitochondria as previously characterized [22]. SOD mimetic treatments were added one hour prior to loading and then maintained during the loading portion of the experiment, about three hours per day, after which explants were placed into fresh growth medium so that each treatment was not present for the unloaded ~20 h each day.

### 2.5. Post-Impact Oxidant Production, Cell Viability, and Mitochondrial Content

For live cell dye oxidation measurements, 24 h after impact, explants were stained in 1 μM dihydroethidium (DHE) in serum free media for 30 min without any SOD mimetics present. This oxidation sensitive dye intercalates into DNA after oxidation and produces a red fluorescence that was visualized by an Olympus FV1000 confocal laser scanning microscope (Olympus Corporation, Tokyo, Japan). Cell viability was confirmed by staining for 30 min with 1 mg/mL Calcein Green AM (Life Technologies, Waltham, MA, USA) for viable cells. This was combined with costaining with 200 nM MitoTracker Deep Red (Life Technologies) for mitochondrial content. Using a 10× objective, within the zone of impact, near the periphery where structural damage from the edge of the impact was near the edge of the image, 10 μm slices were captured through the visible depth of the intact cartilage, ~150 μm on average. Representative images are shown in Figure S1. Viable cell numbers and intensities of both DHE and MitoTracker Deep Red, within live cells only, were then determined throughout that entire volume via custom MATLAB program as previously described [20]. Data shown represent the average live cell intensity of MitoTracker Deep Red. 

### 2.6. Mitochondrial Stress Tests after Repeated Axial Compression

Cartilage from tibial sections subjected to axial compression was harvested from the attached bone, digested using 0.25 mg/mL collagenase/pronase (Sigma, St. Louis, MO, USA), and centrifuged at low speeds to produce enough pelleted chondrocytes for mitochondrial stress tests. Cells were plated at a density of 30,000 cells per well and allowed four days to attach to culture plates with observed plating efficiencies of 50% or greater. Experiments were repeated using explants from four different animals, with no significant animal-to-animal variations observed. Oxygen consumption rates were analyzed according to standard mitochondrial stress test techniques in the Seahorse Bioscience XF96 (Agilent, Santa Clara, CA, USA) and multiple media changes and rinses of each well prior to analysis were used to ensure that no dead cells or debris remained during stress tests as previously described [22]. Reagent injections for the test include 2 μM oligomycin, 0.25 μM carbonyl cyanide-*p*-trifluoromethoxyphenylhydrazone (FCCP), 2 μM rotenone, and 5 μM antimycin A. Basal oxygen consumption rates were taken as the difference between uninhibited oxygen consumption and oxygen consumption after rotenone and antimycin A injection. Maximum oxygen consumption rates were taken as the difference between oxygen consumption after FCCP injection and rates after rotenone and antimycin A injection. Lastly, proton leakage was calculated as the oligomycin-inhibited oxygen consumption rate minus the rate after rotenone and antimycin A injection and then normalized to basal oxygen consumption rate. Following individual runs, wells were trypsinized and counted via hemocytometer with no loss of birefringence noted in these cells, indicating no dead cells were included in the assay.

### 2.7. Statistical Analyses

All statistical analyses were performed using GraphPad Prism 7 (La Jolla, CA, USA). DHE staining was analyzed using student’s *t*-test. All other analyses were two-way ANOVA performed using Dunnett’s post-test to compare multiple treatments. No repeated measures or similar approaches were applied to any matched specimens during analyses and *p* < 0.01 was considered significant.

## 3. Results

### 3.1. SOD Mimetics Provide Protection Against Impact-Induced Loss of Mitochondria

**Figure 2:**
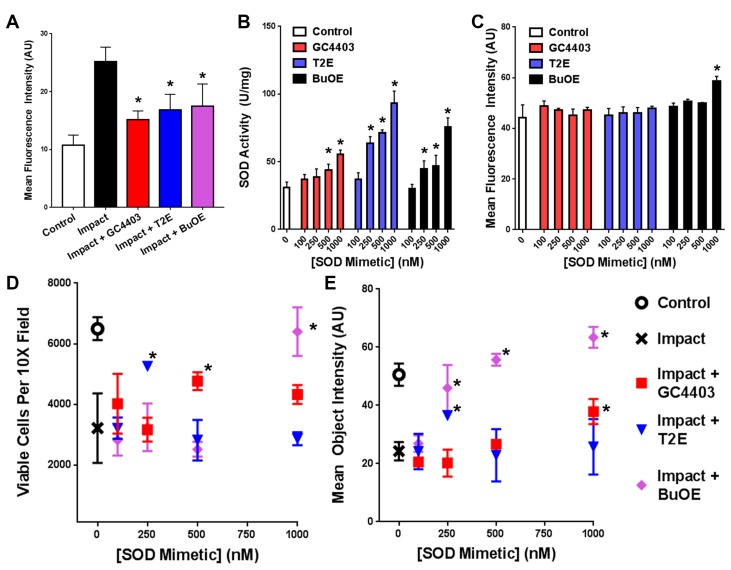
GC4403, T2E, and BuOE provide dose-dependent protection of chondrocytes 24 h after 2 J impacts. (**A**) Previously described [21] increases in DHE staining of chondrocytes within 10× objective confocal images of impacted sites were reproduced and this increase was blunted with treatment by all three mimetics (*n* = 3). (**B**) SOD activity in unloaded primary chondrocytes shows a dose responsive increase with all three SOD mimetics (*n* = 3). (**C**) Mitochondrial staining with MitoTracker Deep Red following mimetic treatments in the absence of loading shows minimal changes in mitochondrial staining with the exception of the 1000 nM dose of BuOE, though many other mimetic treated samples insignificantly trended towards stronger staining than control (*n* = 3). (**D**) Cell counts of Calcein Green AM positive cells from 10× objective confocal images of sites within the impact zone (*n* = 3) show maximal protection by GC4403 between 500 nM and 1000 nM, protection by T2E at 250 nM, and protection by BuOE at 1000 nM. (**E**) Mean intensities of MitoTracker Deep Red from cells in (**A**) show maximal benefit from GC4403 at 1000 nM, protection by T2E at 250 nM, and protection by BuOE peaking at 1000 nM (*n* = 3). * indicate *p* < 0.01 compared to impact tissue without treatment and include both the T2E and BuOE treatments at 250 nM in panel C.

Tissue impacted with a 2 J impact produced greater levels of DHE oxidation than unimpacted controls and this effect could be prevented with addition of 1 μM of any of the SOD mimetics prior to impact (*n* = 3, Figure 2A). All three mimetics increased total SOD activity in a dose dependent manner (*n* = 3, Figure 2B) and only the highest dose of BuOE used, 1000 nM, produced a significant increase in mitochondrial content in the absence of mechanical load (*n* = 3, Figure 2C), though several other groups across the study statistically insignificantly trended higher than controls. Explants were assessed for chondrocyte viability within zones of impact alongside mitochondrial content at identical loci, representative images shown in Figure S1. Cell viability measurements indicated a dose-dependent protective effect from the impact injury for each of the three mimetics (*n* = 3, Figure 2D). GC4403 shows its greatest benefit to viability at 500 nM, T2E at 250 nM, and BuOE at 1 μM, with both GC4403 and T2E demonstrating a dose dependent response that appears to be biphasic, increasing and then decreasing. Pilot studies of all three compounds above this highest dose agreed with previous studies suggesting a ceiling for use above 1 μM [27,28,29]. Costaining for mitochondrial content indicates modest dose-dependent protection by T2E, but much more substantial protection by BuOE at each dose (*n* = 3, Figure 2E).

### 3.2. BuOE Protects against Losses in Mitochondria after Repeated Axial Compression

**Figure 3:**
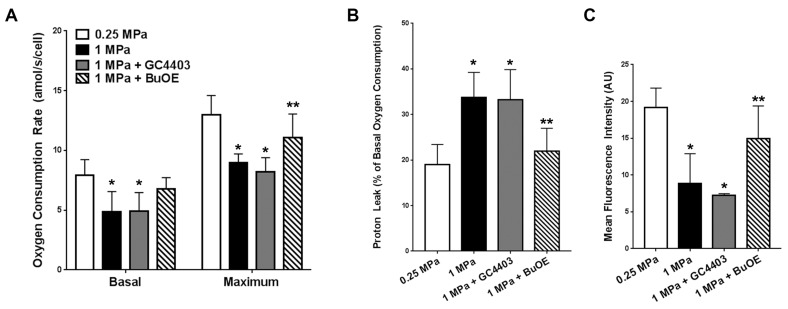
BuOE but not GC4403 prevents indications of mitochondrial dysfunction after repetitive compressive loading. (**A**) Mitochondrial stress test-derived basal and maximum oxygen consumption rates of chondrocytes extracted from the loaded portions of healthy 0.25 MPa loaded explants and injurious 1.0 MPa loaded explants with or without GC4403 or BuOE (*n* = 4). (**B**) Proton leak by extracted chondrocytes as a percentage of the basal activity by each sample (*n* = 4). (**C**) Overall mean fluorescence intensity of confocal images taken of cross sections of loaded portions of each explant staining with MitoTracker Deep Red (*n* = 4). * indicate *p* < 0.01 compared to 0.25 MPa loading and ** indicate *p* < 0.01 compared to 1 MPa loading without treatment.

After the seven day axial loading experiment, functional decreases in mitochondria were observed via XF96 including 40% losses in basal respiration and maximal respiration with overload that were prevented with BuOE but not GC4403 (Figure 3A). Overloading also elicited an increase in proton leak expressed as a percentage of basal respiration (Figure 3B). These indications of mitochondrial membrane damage were decreased by BuOE but not GC4403 (Figure 3B). Similar MitoTracker Deep Red staining to the impact study revealed a decrease in mitochondrial staining with repeated axial compression that was prevented by BuOE but not GC4403 (Figure 3C).

## 4. Discussion

The primary finding of this work is that a tightly dose-dependent benefit for mitochondria is seen when applying all three SOD mimetics after impact injuries and that this benefit is not observed uniformly in a chronic overloading scenario, suggesting that distinctions between redox biological conditions resulting from impulse and chronic loading are necessary. This is unsurprising given that disruptions in MnSOD are already associated with repeated mechanical overload as well as osteoarthritis [23,24,25,26] and inflammatory signals commonly associated with injury and arthritis can alter MnSOD content [32]. These studies suggest a complex interplay between redox biology, mechanics, and the progressively degrading environment of PTOA. Our study is the first demonstration to our knowledge that small molecular weight SOD mimetics can provide a tangible benefit to protecting mitochondrial content within otherwise normal tissue after mechanical impact. The degree of this protection and the consistency of some observed benefit from each of the three mimetics after impact strongly support the hypothesis that superoxide can contribute to chondrocyte mitochondrial damage in mechanical impact scenarios.

By contrast, the lipophilic SOD mimetic BuOE provided protection from loss of mitochondria when applied daily throughout seven days of repeated axial overload while the more hydrophilic mimetic GC4403 provided no protection. Pilot experiments using higher doses of GC4403 provided no additional benefit in the seven day overloading scenario, suggesting that the lack of protection observed from this compound was not as a result of inadequate SOD supplementation. Rather, we believe these experiments indicate that while impact injuries cause sharp increases in superoxide production, chronic overloading may be causing more complex changes in redox biology centering around the lipid compartment of articular chondrocytes. Mitochondrial membranes may therefore represent a critical locus of damage to articular chondrocyte mitochondria.

The distinctions between outcomes after impact and axial loading have important implications for redox biological approaches to future development of therapies for articular cartilage. In an acute impact setting, our results strongly support the hypothesis that mechanical impact causes aberrant one-electron reductions of oxygen and that this contributes to cell death. Thus, our data support the use of SOD acutely; however, increases in protection by the aqueous mimetics are lost at higher doses possibly suggesting a more nuanced redox environment where increasing superoxide removal becomes detrimental, possibly as a result of too much hydrogen peroxide product at high doses of GC4403 and T2E. Alternatively, in the repeated overload scenario there is a strong coherence between protection of mitochondrial function with BuOE, our prior results demonstrating the benefits of NAC and MitoQ in this setting [19,22], and existing literature showing critical interactions between lipid peroxidation and the glutathione system [33,34]. These studies imply that oxidative damage to mitochondrial lipids resulting from mechanical injury or other stresses feeds forward into general oxidative stress responses mediated by the intracellular thiol systems. Throughout PTOA pathogenesis as inflammation ebbs and flows, tissue begins to degrade, and local mechanics become further disrupted, chronic indications of lipid peroxidation and the involvement of intracellular thiols are noted at many stages [19,20,21,22,23,24,25,26,33,34]. This suggests that future attempts to improve the chronic redox environment within chondrocytes in response to mechanical overload will need to account for the state of the tissue, from healthy to arthritic, and the nature of the mechanical overload itself.

Several limitations to this study are worth noting. First, because the mimetics used freely diffuse throughout tissue and chondrocytes, it is impossible to discard the possibility of extracellular effects from these treatments. Articular cartilage possesses high levels of extracellular SOD (SOD3) which is known to decrease with disease in humans [35] and plays an important role in maintenance of healthy tissue [36]. Our interest in this study was to examine the effect of augmenting superoxide removal upon a specific pathology, cell death, and loss of mitochondrial content, but it is worth noting that clinical trials of an extracellular version of the native SOD, orgotein, injected intraarticularly into the knee after development of disease originally showed some promise but it is unknown whether these results have been extended further [37]. Second, specific cell death pathways—i.e., necrosis vs. apoptosis—after impact or overload were not queried in favor of studying the mitochondrial status of the remaining viable cells. Future studies of protection by SOD may reveal important features of how these cell death pathways function after mechanical injury. Third, T2E was not evaluated in the chronic setting, but this was avoided for several reasons. Repeated overloading experiments involved daily sustained exposures to the SOD mimetics. GC4403 and BuOE can be given at the same concentration and provided some protection after a broader range of concentrations, suggesting that relatively high daily exposures could be well tolerated by healthy tissue. Impact data suggested that T2E had a fairly narrow treatment window for protection. Thus, repeated exposures daily for seven days may yield less predictable results or difficult results to interpret, a significant concern in the chronic setting where throughput is much lower. Given that stronger benefits after impact were observed with the BuOE analog, and that BuOE and T2E have similar chemical activity, only BuOE and GC4403 were applied in the chronic overload setting. The fourth limitation of this work is that native enzymes were not utilized in this study. The three SOD isoforms each have complex functions, interactions with other proteins, and myriad regulatory structures in addition to a higher rate constant than any of the mimetics utilized here. We have chosen to avoid disruptions in the native enzyme levels in order to focus directly on the effect of supplementing superoxide removal throughout normal, healthy tissue and chondrocytes. Future work will be focused on exploring more site specific features of the redox biology described.

## 5. Conclusions

In summary, the results presented here demonstrate that excess superoxide is likely contributing to cell death observed with impact injury but losses in mitochondrial function observed with chronic overloading or impact are not uniformly responsive to increasing superoxide removal capacity. Instead, repeated overload appears to induce mitochondrial changes through redox events in the lipid compartment that can be interrupted by BuOE. This may indicate that ongoing translational efforts to block the earliest phases of PTOA should be focused on events within the lipid compartment. Future studies of the mitochondrial pathology described here will focus on the oxidation of mitochondrial lipid components in order to determine what mechanism leads to mitochondrial dysfunction. While the sequence of drug administration followed by acute impact is impossible in a post-traumatic setting where patients present hours or even days after injury, improved understanding of the delicate redox environment of the articular chondrocyte after impact and repeated axial compression may lead to better target identification for prevention of PTOA.

## Supplementary Materials

The following are available online at www.mdpi.com/2076-3921/6/4/98/s1, Figure S1: Representative MitoTracker Staining for Quantitation of Live-Cell Mitochondrial Content.
