# Supplementary material for: Differential Effects of Superoxide Dismutase Mimetics after Mechanical Overload of Articular Cartilage
Source: Antioxidants (Basel). 2017 Nov 30;6(4):98. doi: 10.3390/antiox6040098 (PMC5745508; doi:10.3390/antiox6040098)
Supplement: Supplementary file 1 [file antioxidants-06-00098-s001.pdf]

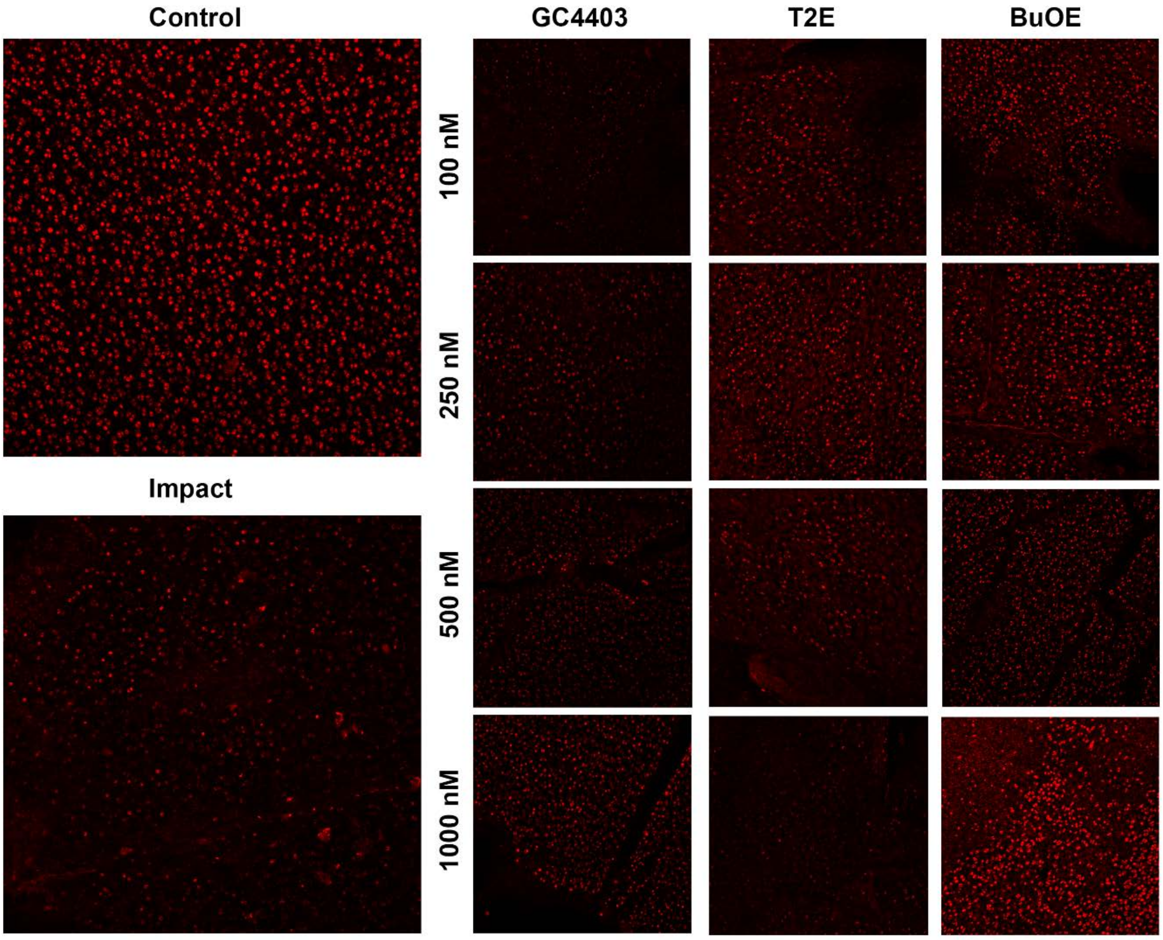

Figure S1. Representative MitoTracker staining for quantitation of live-cell mitochondrial content.
